# Hemoglobin Trajectories After Metformin Initiation Compared With Dipeptidyl Peptidase-4 Inhibitors: A Real-World Cohort Study

**DOI:** 10.7759/cureus.109639

**Published:** 2026-05-25

**Authors:** Yasuko Morita, Kazuhiko Sakaguchi, Shun-ichiro Asahara, Wataru Ogawa, Kenji Sugawara

**Affiliations:** 1 Division of Diabetes and Endocrinology, Department of Internal Medicine, Kobe University Graduate School of Medicine, Kobe, JPN; 2 Division of Community Medicine and Medical Education, Department of Social/Community Medicine and Health Science, Kobe University Graduate School of Medicine, Kobe, JPN; 3 Division of Metabolic Diseases, Department of Translational Medical Science, Kobe University Graduate School of Medicine, Kobe, JPN

**Keywords:** anemia, electronic medical records, hemoglobin, metformin, type 2 diabetes

## Abstract

Background

Metformin is associated with vitamin B_12_ deficiency, and we recently reported latent iron and copper deficiency among metformin users, suggesting a potential contribution to anemia risk. However, real-world evidence on hemoglobin trajectories after metformin initiation remains limited. We evaluated short- and long-term hemoglobin changes after metformin initiation compared with dipeptidyl peptidase-4 (DPP-4) inhibitors in Japanese patients with type 2 diabetes.

Methods

This single-center retrospective cohort study used electronic medical record data from Kobe University Hospital from January 2014 to June 2025. Short-term hemoglobin changes were defined as changes from Day 0 to Day 180, and long-term trajectories were evaluated from Day 0 to Day 1,826 (five years). Patients initiating metformin or a DPP-4 inhibitor were selected using predefined eligibility and exclusion criteria and matched by propensity scores. Hemoglobin changes over 180 days and up to five years were analyzed using multiple regression and mixed-effects models.

Results

The short-term cohort included 72 matched pairs, and the long-term cohort included 182 matched pairs. Hemoglobin decreased from 14.74 ± 0.22 to 14.39 ± 0.23 g/dL (mean ± SEM) from Day 0 to Day 180 in the metformin group, whereas no significant change was observed in the DPP-4 inhibitor group. Pre-initiation hemoglobin change, but not metformin dose, was independently associated with subsequent hemoglobin change. Over five years, annual hemoglobin slopes did not differ significantly between groups (-0.0572 vs. -0.0725 g/dL/year, p = 0.31). Older patients showed more negative annual hemoglobin slopes in both groups.

Conclusions

Metformin initiation was associated with a short-term hemoglobin decrease, whereas excess long-term decline was not observed in this cohort. Long-term changes were modest and age-related, supporting continued monitoring in older patients.

## Introduction

Metformin has been used for more than 60 years and remains one of the most widely prescribed glucose-lowering agents for type 2 diabetes worldwide. Its glucose-lowering action is mainly attributed to suppression of hepatic gluconeogenesis, partly through effects on mitochondrial energy metabolism [1,2]. However, metformin also acts beyond the liver, particularly in the gastrointestinal tract, where it influences gut microbiota, bile acid metabolism, intestinal glucose dynamics, and enterocyte function [2-4]. Despite its long-standing clinical use, the mechanisms underlying these glucose-lowering and pleiotropic actions remain incompletely understood [1,2].

Metformin is known to be associated with vitamin B_12_ deficiency, which may contribute to anemia in susceptible patients [5,6]. In addition, we previously reported in a cross-sectional analysis that metformin treatment was associated with altered metal dynamics, including findings suggestive of latent iron and copper deficiencies [7]. Together, these findings raise the possibility that metformin may influence hematologic status. A previous analysis of randomized clinical trial data suggested that metformin use may be associated with early anemia risk and hemoglobin decline after treatment initiation, without evidence of progressive long-term decline specific to metformin [8].

However, longitudinal real-world data evaluating hemoglobin trajectories after metformin initiation are limited, particularly in Asian patients. Randomized trials provide high internal validity but may not fully reflect real-world prescribing patterns, treatment continuation, laboratory monitoring, and patient characteristics. Electronic medical record-based real-world data can therefore provide complementary evidence regarding the hematologic safety of metformin in routine diabetes care.

In the present study, we used electronic medical record data from a Japanese university hospital to compare short- and long-term hemoglobin changes after initiation of metformin or dipeptidyl peptidase-4 (DPP-4) inhibitors. The primary objective of this study was to compare short-term and long-term hemoglobin trajectories between patients initiating metformin and those initiating DPP-4 inhibitors in routine clinical practice. We evaluated hemoglobin change during the first 180 days and annual hemoglobin trajectories over five years using propensity score-matched cohorts and linear mixed-effects models.

## Materials and methods

Study design and data source

This was a single-center, retrospective, real-world cohort study using electronic medical record data from Kobe University Hospital, Kobe, Japan. The study was conducted in accordance with the Declaration of Helsinki and its amendments and was approved by the Clinical Research Ethics Committee of Kobe University Graduate School of Medicine (approval no. B250244; approval date: March 17, 2026). The study was also permitted by the Director of Kobe University Hospital on March 19, 2026. The requirement for written informed consent was waived because of the retrospective nature of the study, and the study was conducted using anonymized or de-identified clinical data. Information on the study was disclosed to eligible patients using an opt-out approach.

A feasibility check was conducted before formal data extraction using SIMPRESEARCH® (4DIN Ltd., Tokyo, Japan). In the present analysis, patients with a history of receiving metformin or a DPP-4 inhibitor between January 1, 2014, and June 30, 2025, were identified from electronic medical records. Laboratory measurements were performed as part of routine clinical practice at a single institution.

Study population

From the electronic medical records, 6,852 metformin users and 13,025 DPP-4 inhibitor users were initially identified. For the short-term cohort, patients were required to have a prescription for the same drug within 180 ± 30 days from the initial prescription date, with treatment coverage of at least 80% during the first 180 days. Treatment coverage was defined as the proportion of days covered by prescription records during the observation period. Dose adjustment within the same drug was considered treatment continuation, whereas switching to a different drug class was considered treatment discontinuation. Multiple prescriptions on the same day were treated as continuous treatment. Patients with missing hemoglobin values at predefined time points were excluded from the analysis. Baseline variables were obtained from measurements at or closest to Day 0 within the predefined baseline window. After applying the treatment continuation criterion, 1,745 metformin users and 2,751 DPP-4 inhibitor users remained. Patients were excluded if they had hospitalization during the observation period, unavailable hemoglobin measurements at Day -90, Day 0, or Day 180, anemia at baseline, severe renal impairment at baseline, defined as an estimated glomerular filtration rate (eGFR) < 30 mL/min/1.73 m², use of anemia-related therapies, concomitant use of metformin and a DPP-4 inhibitor, or age < 20 years.

For the long-term cohort, patients were required to have hemoglobin measurements at Day 0 and within Day -90 ± 30 days. To estimate individual hemoglobin slopes, all available hemoglobin measurements from the initial prescription date up to five years were used. Patients were excluded if they had hospitalization during the observation period, age < 20 years, anemia at baseline, severe renal impairment at baseline, defined as eGFR < 30 mL/min/1.73 m², insufficient treatment continuation, use of anemia-related therapies from Day -120 to 30 days after the last hemoglobin measurement used for slope estimation, or duplicate records. Insufficient treatment continuation was defined as failure to maintain prescription coverage for the index drug during follow-up based on prescription records.

After these exclusions, 93 metformin users and 96 DPP-4 inhibitor users were eligible for propensity score matching in the short-term cohort, and 203 metformin users and 224 DPP-4 inhibitor users were eligible for propensity score matching in the long-term cohort. Propensity score matching was performed separately for each cohort using age, sex, and baseline eGFR. One-to-one nearest-neighbor matching was performed without replacement, with a caliper width of 0.2 standard deviations of the logit of the propensity score, yielding 72 matched pairs in the short-term cohort and 182 matched pairs in the long-term cohort.

Definition of anemia

Anemia was defined using sex- and age-specific hemoglobin thresholds based on Japanese criteria for renal anemia: hemoglobin < 13.5 g/dL for men aged < 60 years, < 11.5 g/dL for women aged < 60 years, < 12.0 g/dL for men aged 60-69 years, < 10.5 g/dL for women aged 60-69 years, < 11.0 g/dL for men aged ≥ 70 years, and < 10.5 g/dL for women aged ≥ 70 years [9]. Patients meeting the anemia criterion at Day 0 were excluded from the main analyses.

Outcomes

The primary short-term outcome was the change in hemoglobin from Day 0 to Day 180. The long-term outcome was the annual rate of hemoglobin change over five years after treatment initiation. Additional analyses examined factors associated with hemoglobin change from Day 0 to Day 180 and age-stratified hemoglobin trajectories in patients aged <65 years and ≥65 years.

Linear mixed-effects models

Longitudinal hemoglobin trajectories were modeled using linear mixed-effects models. Time was calculated as days from the index date, converted to years, and used as a continuous variable in the mixed-effects models. For each treatment group, hemoglobin values were modeled as a function of time from the index date, with a fixed intercept, a fixed slope representing the annual hemoglobin change, and a patient-specific random intercept. Only hemoglobin measurements from Day 0 to Day 1,826 were included. Between-group differences in annual hemoglobin change were assessed by adding a group-by-time interaction term to the model, with the DPP-4 inhibitor group as the reference.

Statistical analysis

Between-group comparisons were performed using Welch’s t-test or the Mann-Whitney U test for continuous variables and the chi-square test or Fisher’s exact test for categorical variables. Within-group comparisons between Day 0 and each follow-up time point (Day 60, Day 120, and Day 180) were performed using the paired t-test or the Wilcoxon signed-rank test. Multiple linear regression was used to identify factors associated with hemoglobin change from Day 0 to Day 180. Candidate covariates included age, sex, treatment group, eGFR at Day 0, HbA1c at Day 0, and hemoglobin change from Day -90 to Day 0. In the metformin group, metformin daily dose was additionally included. All statistical analyses were performed using R software, version 4.5.3 (The R Foundation for Statistical Computing, Vienna, Austria), with the MatchIt, lme4, and lmerTest packages [10-12]. A two-sided p-value < 0.05 was considered statistically significant. Given the exploratory nature of the additional analyses, formal correction for multiple comparisons was not applied.

## Results

Short-term hemoglobin change after treatment initiation

**Figure 1:**
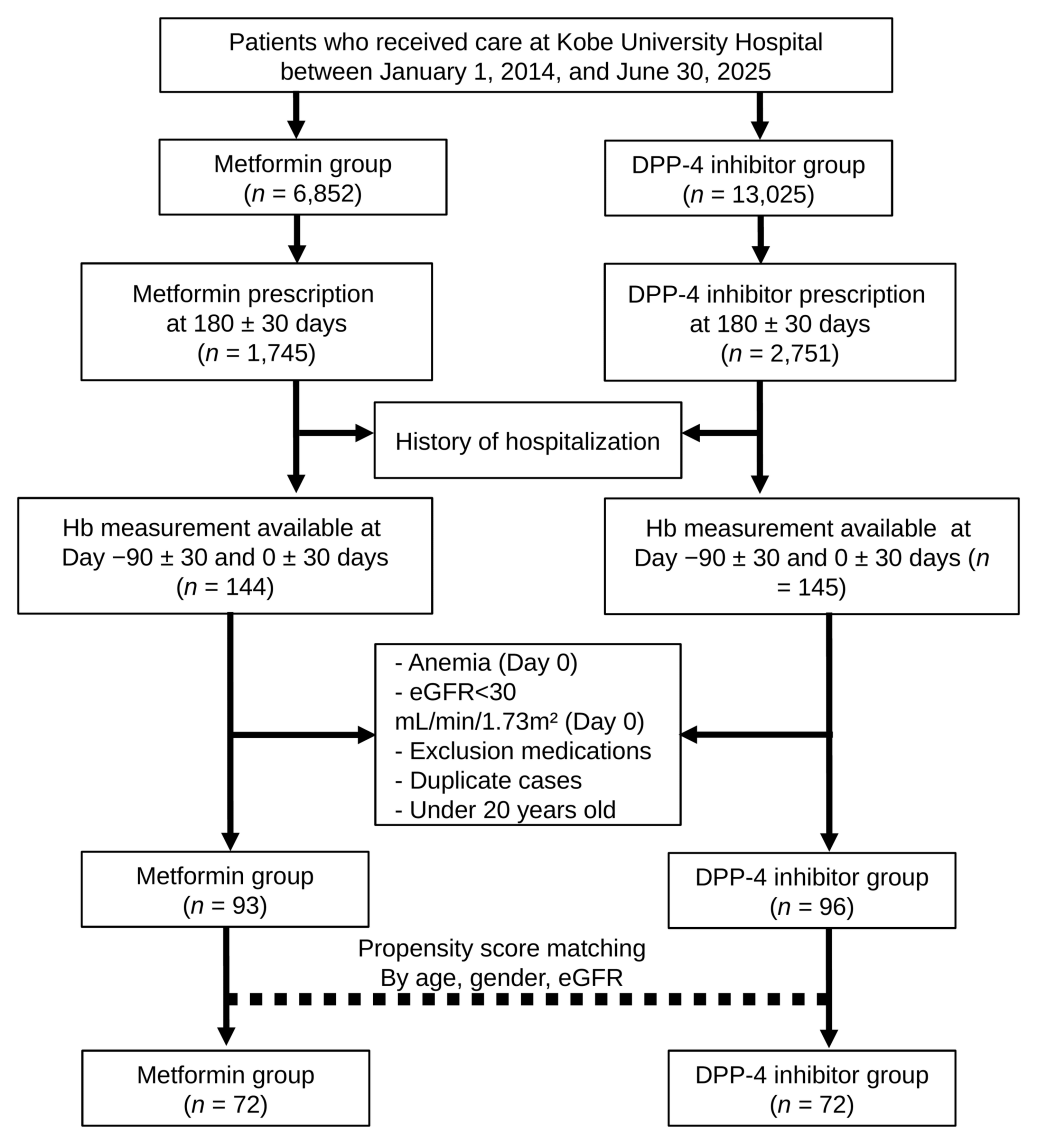
Flowchart of patient selection for the short-term cohort Patients initiating metformin or a DPP-4 inhibitor were identified from electronic medical records and selected according to treatment continuation, hemoglobin measurement availability, exclusion criteria, and propensity score matching. DPP-4, dipeptidyl peptidase-4; eGFR, estimated glomerular filtration rate

After applying the eligibility and exclusion criteria, propensity score matching yielded 72 matched pairs in the short-term cohort (Figure 1).

Baseline characteristics did not differ significantly between the metformin and DPP-4 inhibitor groups after matching (Table 1).

**Table 1:** Baseline characteristics of the short-term cohort after propensity score matching Data are presented as mean ± SD or median (IQR) for continuous variables, as numbers for categorical variables. p-values indicate comparisons between metformin and DPP-4 inhibitor groups using the Mann-Whitney U test for continuous variables, and Pearson's chi-squared test for categorical variables. DPP-4i, dipeptidyl peptidase-4 inhibitor; eGFR, estimated glomerular filtration rate; HbA1c, glycated hemoglobin

Variable	Metformin group (n = 72)	DPP-4 inhibitor group (n = 72)	Statistic	p-value
Age (years)	65.5 (56.8, 69.3)	64.0 (58.0, 71.3)	U = 2524	0.787
Sex (male/female)	44/28	45/27	χ² = 0.000	1.000
Metformin daily dose (mg/day)	506.9 ± 93.6	-	-	-
HbA1c (%)	7.6 (7.1, 8.1)	7.4 (6.9, 7.8)	U = 2919	0.143
eGFR (mL/min/1.73 m²)	74.6 (59.1, 85.2)	71.8 (61.5, 81.5)	U = 2702	0.662

**Figure 2:**
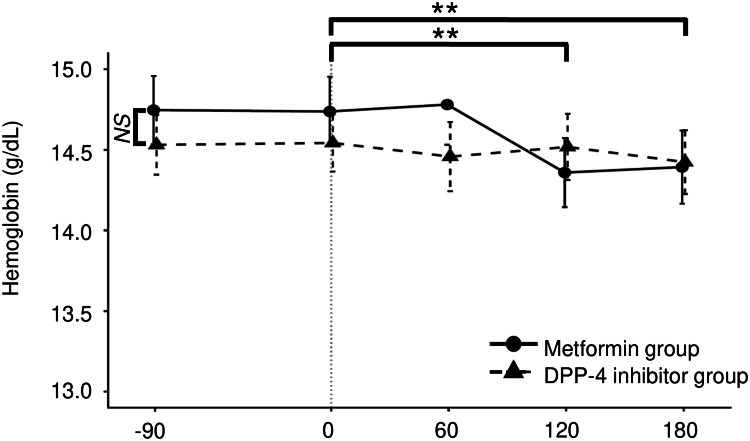
Short-term hemoglobin changes after treatment initiation Short-term changes in hemoglobin from Day -90 to Day 180 are shown for the metformin and DPP-4 inhibitor groups. Data are presented as mean ± SEM. **p <0.01; NS denotes not significant. DPP-4, dipeptidyl peptidase-4

The mean age was 62.4 ± 12.0 years in the metformin group and 62.7 ± 12.5 years in the DPP-4 inhibitor group (mean ± SD). In the metformin group, the mean daily dose was 506.9 ± 93.6 mg/day at Day 0 and 684.0 ± 363.4 mg/day at Day 180 (mean ± SD). In this cohort, hemoglobin levels in the DPP-4 inhibitor group remained largely stable from Day 0 to Day 180. In contrast, the metformin group showed a modest but statistically significant decrease in hemoglobin over the same period (Day 0, 14.74 ± 0.22 g/dL; Day 180, 14.39 ± 0.23 g/dL; mean ± SEM; p <0.001). In the DPP-4 inhibitor group, hemoglobin was 14.54 ± 0.18 g/dL at Day 0 and 14.42 ± 0.20 g/dL at Day 180 (mean ± SEM; p = 0.14) (Figure 2).

Multiple linear regression analysis in the combined short-term cohort showed that age, treatment group, and hemoglobin change from Day -90 to Day 0 were independently associated with hemoglobin change from Day 0 to Day 180 (Table 2).

**Table 2:** Multiple linear regression analysis for hemoglobin change (Day 0 to Day 180) β, standardized coefficient; CI, confidence interval; DPP-4i, dipeptidyl peptidase-4 inhibitor; eGFR, estimated glomerular filtration rate; HbA1c, glycated hemoglobin; Met, metformin; ΔHb, change in hemoglobin

Parameter	β	p	95% CI
Age (years)	-0.0099	0.0294	-0.0188, -0.0011
Sex (male/female)	0.1273	0.2343	-0.0816, 0.3362
Group: DPP-4i vs. Met	-0.2197	0.0354	-0.4223, -0.0171
eGFR at Day 0 (mL/min/1.73 m²)	-0.0005	0.8533	-0.0059, 0.0048
HbA1c at Day 0 (%)	-0.0305	0.5499	-0.1301, 0.0691
ΔHb (Day -90 to Day 0) (g/dL)	-0.4396	<0.001	-0.6196, -0.2596
R²	0.2202		
Adjusted R²	0.1858		

The strongest association was observed for hemoglobin change before treatment initiation. In the analysis restricted to the metformin group, metformin daily dose was not independently associated with hemoglobin change, whereas hemoglobin change from Day -90 to Day 0 remained significantly associated with subsequent hemoglobin change (Table 3).

**Table 3:** Multiple linear regression analysis for hemoglobin change (Day 0 to Day 180) in only metformin group β, standardized coefficient; CI, confidence interval; DPP-4i, dipeptidyl peptidase-4 inhibitor; eGFR, estimated glomerular filtration rate; HbA1c, glycated hemoglobin; Met, metformin; ΔHb, change in hemoglobin

Parameter	β	p	95% CI
Age (years)	-0.0101	0.1101	-0.0224, 0.0021
Sex (male/female)	0.1212	0.4295	-0.1776, 0.4200
Metformin daily dose (mg/day)	0.0004	0.6489	-0.0012, 0.0019
eGFR at Day 0 (mL/min/1.73 m²)	-0.0037	0.2821	-0.0102, 0.0029
HbA1c at Day 0 (%)	0.1275	0.0881	-0.0168, 0.2718
ΔHb (Day -90 to Day 0) (g/dL)	-0.5281	<0.001	-0.8078, -0.2484
R²	0.2343		
Adjusted R²	0.1637		

Long-term hemoglobin trajectories

**Figure 3:**
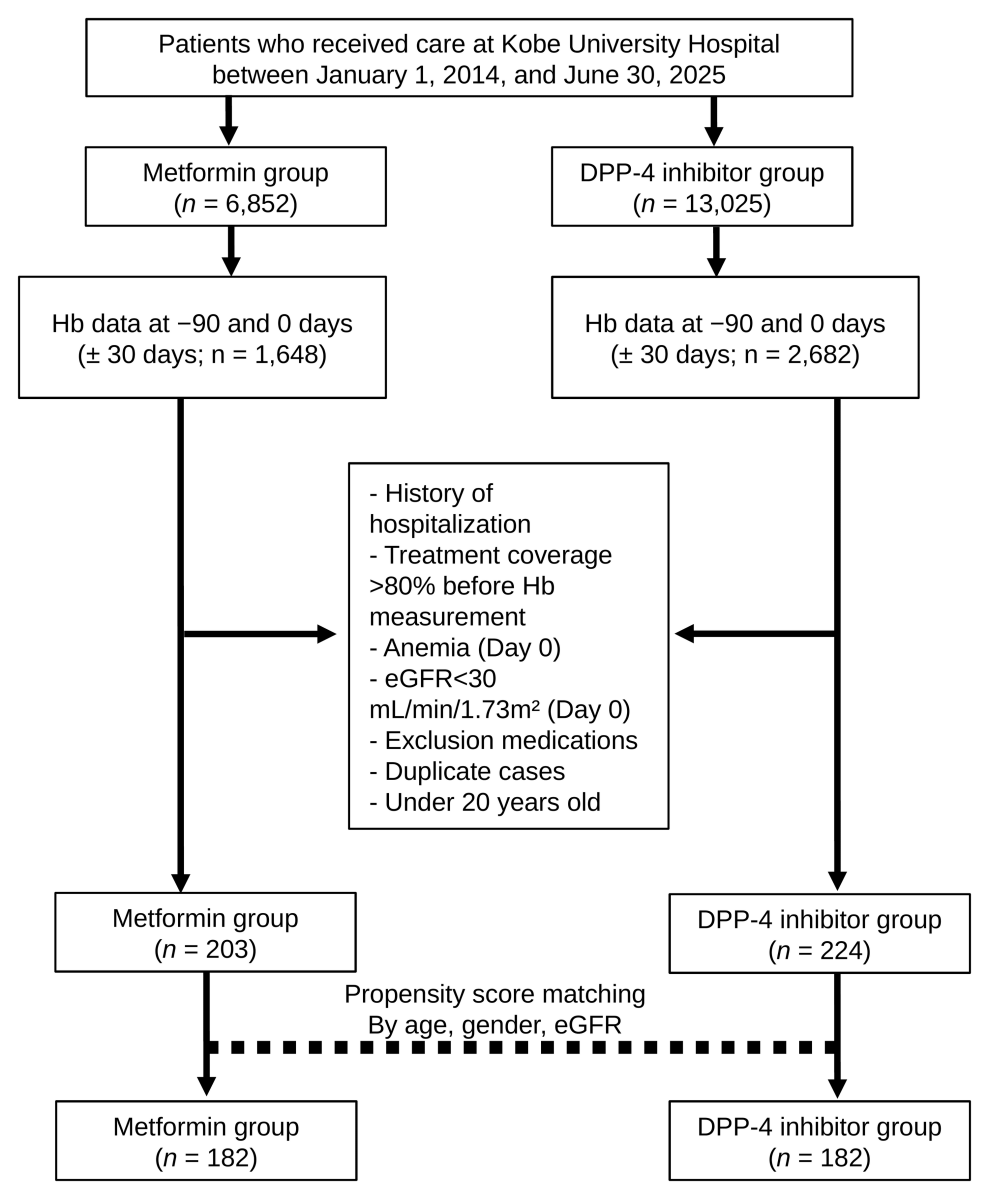
Flowchart of patient selection for the long-term cohort Patients initiating metformin or a DPP-4 inhibitor were identified from electronic medical records and selected according to hemoglobin measurement availability, treatment continuation, exclusion criteria, and propensity score matching. DPP-4, dipeptidyl peptidase-4; eGFR, estimated glomerular filtration rate

After applying the eligibility and exclusion criteria, propensity score matching yielded 182 matched pairs in the long-term cohort (Figure 3).

After matching, baseline characteristics did not differ significantly between the metformin and DPP-4 inhibitor groups (Table 4).

**Table 4:** Baseline characteristics of the long-term cohort after propensity score matching Data are presented as mean ± SD or median (IQR) for continuous variables, as numbers for categorical variables. p-values indicate comparisons between metformin and DPP-4 inhibitor groups using the Mann-Whitney U test for continuous variables, and Pearson's chi-squared test for categorical variables. DPP-4i, dipeptidyl peptidase-4 inhibitor; eGFR, estimated glomerular filtration rate; HbA1c, glycated hemoglobin

Variable	Metformin group (n = 182)	DPP-4 inhibitor group (n = 182)	Statistic	p-value
Age (years)	63.0 (52.3, 71.0)	64.0 (55.0, 71.8)	U = 15629	0.353
Sex (male/female)	106/76	110/72	χ² = 0.182	0.670
Metformin daily dose (mg/day)	565.3 ± 242.2	-	-	-
HbA1c (%)	7.4 (6.9, 8.1)	7.3 (6.7, 7.8)	U = 17810	0.151
eGFR (mL/min/1.73 m²)	73.4 (59.4, 84.1)	71.3 (60.6, 82.1)	U = 17235	0.503

**Figure 4:**
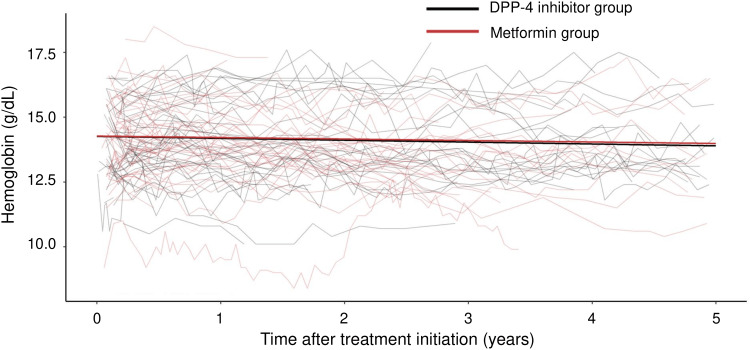
Long-term hemoglobin trajectories after treatment initiation Long-term hemoglobin trajectories over up to five years are shown. Thin black lines indicate individual patient trajectories in the DPP-4 inhibitor group, and thin red lines indicate individual patient trajectories in the metformin group. Bold black and red lines indicate group-level trajectories estimated by linear mixed-effects models for the DPP-4 inhibitor and metformin groups, respectively. DPP-4, dipeptidyl peptidase-4

Longitudinal analysis over up to five years showed no significant difference in annual hemoglobin slope between the two groups. The estimated annual hemoglobin change was -0.0572 g/dL/year in the metformin group and -0.0725 g/dL/year in the DPP-4 inhibitor group (p = 0.31) (Figure 4).

These findings suggest that, in this cohort, metformin initiation was not associated with excess long-term hemoglobin decline compared with DPP-4 inhibitor initiation.

Age-related differences in long-term hemoglobin trajectories

**Figure 5:**
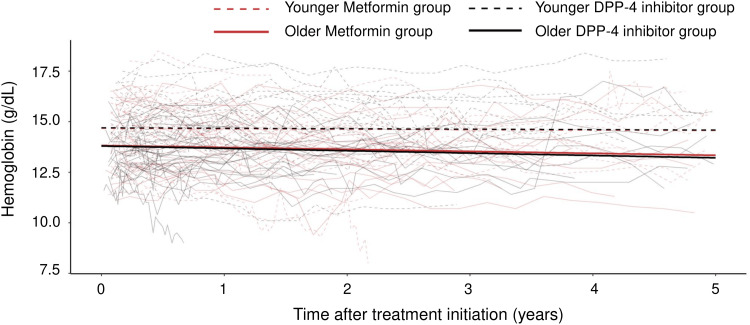
Long-term hemoglobin trajectories by age group Long-term hemoglobin trajectories are shown according to age group. Red and black lines indicate the metformin and DPP-4 inhibitor groups, respectively. Dashed and solid lines indicate younger (< 65 years) and older (≥ 65 years) patients, respectively. Thin lines indicate individual patient trajectories, and bold lines indicate group-level trajectories estimated by linear mixed-effects models. DPP-4, dipeptidyl peptidase-4

When patients were stratified by age, patients aged ≥ 65 years had a more negative annual hemoglobin slope than those aged < 65 years in both treatment groups. In the metformin group, the estimated annual hemoglobin change was -0.0218 g/dL/year in younger patients and -0.0970 g/dL/year in older patients (p = 1.405 × 10^-3^). In the DPP-4 inhibitor group, the estimated annual hemoglobin change was -0.0224 g/dL/year in younger patients and -0.1171 g/dL/year in older patients (p = 5.805 × 10^-6^) (Figure 5).

## Discussion

In this single-center Japanese real-world cohort study, we compared short- and long-term hemoglobin trajectories after initiation of metformin or DPP-4 inhibitors. Metformin initiation was associated with a small but statistically significant decrease in hemoglobin during the first 180 days, whereas metformin dose was not independently associated with hemoglobin change within the metformin group. In contrast, longitudinal analysis over up to five years showed no significant difference in annual hemoglobin decline between the metformin and DPP-4 inhibitor groups. In both treatment groups, older patients had a more negative annual hemoglobin slope than younger patients.

Metformin is known to be associated with vitamin B_12_ deficiency [5,6]. We also previously reported that metformin treatment was associated with altered metal dynamics, including findings suggestive of latent iron and copper deficiencies [7]. A previous analysis of randomized clinical trial data suggested that metformin use may be associated with early anemia risk and hemoglobin decline after treatment initiation [8]. The present real-world findings are consistent with these reports in that hemoglobin decreased during the first six months after metformin initiation. However, the absolute magnitude of the decrease was small, and the absence of an association with metformin dose suggests that the short-term hemoglobin decline may not be explained solely by a direct dose-dependent hematologic effect of metformin.

Multivariable regression analysis showed that hemoglobin change from Day -90 to Day 0 was strongly associated with subsequent hemoglobin change from Day 0 to Day 180. Patients requiring initiation of an additional glucose-lowering medication may have relatively marked hyperglycemia. In such patients, osmotic diuresis and changes in hydration status associated with hyperglycemia may influence hemoglobin and hematocrit values. A previous clinical study also suggested that glycemic status and metformin administration may affect red blood cell indices and oxidative stress markers [13]. Therefore, pretreatment hemoconcentration or hyperglycemic status may partly explain the early decrease in hemoglobin after treatment initiation in real-world practice. This mechanism may not be specific to metformin and could partly reflect clinical changes accompanying initiation of oral glucose-lowering therapy.

The long-term analysis did not show evidence of excess hemoglobin decline associated with metformin use in this cohort. Over up to five years, the annual rate of hemoglobin change did not differ significantly between the metformin and DPP-4 inhibitor groups. The estimated slopes in both groups were small. A longitudinal Swedish cohort of older adults reported age-related hemoglobin decline over 18 years, with annual changes of approximately -0.053 g/dL/year in healthy men and -0.005 g/dL/year in healthy women [14]. Although differences in population characteristics and analytical methods should be considered, the long-term slopes observed in the present study appear broadly compatible with modest age-related hemoglobin changes rather than progressive drug-induced anemia.

In the age-group analysis, annual hemoglobin slopes did not differ significantly between the metformin and DPP-4 inhibitor groups among either patients aged < 65 years or those aged ≥ 65 years. In contrast, older patients showed a greater decline in hemoglobin than younger patients in both treatment groups. This finding suggests that age, rather than metformin exposure alone, is an important determinant of long-term hemoglobin trajectories. In clinical practice, periodic hemoglobin monitoring remains appropriate in older patients with type 2 diabetes, regardless of the glucose-lowering medication used.

This study has several strengths. We used electronic medical record data collected over more than 10 years, defined treatment initiation as the index date, and evaluated both short-term and long-term hemoglobin changes. We also used an active comparator design and propensity score matching to reduce baseline differences between treatment groups. In addition, longitudinal hemoglobin trajectories were assessed using mixed-effects models, allowing repeated hemoglobin measurements over time to be incorporated into the analysis.

Several limitations should also be acknowledged. First, this was a single-center retrospective study, and the sample size after applying strict eligibility criteria and propensity score matching was relatively small for a real-world data analysis, which may have limited statistical power and the precision of estimated effects. Second, measurements of iron, ferritin, transferrin saturation, copper, and vitamin B_12_ were not available in sufficient numbers, preventing evaluation of nutritional or metal-related pathways. Third, although DPP-4 inhibitors were used as an active comparator, differences in clinical indications and prescribing patterns between metformin and DPP-4 inhibitors may remain. Fourth, direct measures of hydration status or plasma volume were unavailable, limiting evaluation of whether early hemoglobin changes reflected glycemic improvement, hemoconcentration, or other clinical factors. Fifth, the short-term and long-term cohorts were not identical populations because separate eligibility criteria were applied for each analysis.

## Conclusions

In this Japanese real-world cohort study using electronic medical record data, metformin initiation was associated with a small decrease in hemoglobin during the first six months compared with DPP-4 inhibitor initiation. This short-term change appeared to be influenced by the pre-initiation hemoglobin trajectory, and metformin dose was not independently associated with hemoglobin change within the metformin group. Over up to five years, metformin was not associated with excess long-term hemoglobin decline compared with DPP-4 inhibitors. These findings suggest that, in this single-center retrospective cohort, metformin was not associated with clinically meaningful progressive hemoglobin decline compared with DPP-4 inhibitors, although regular hemoglobin monitoring should be considered in older patients.
